# Supplementary material for: The Construction and Exploration of a Comprehensive MicroRNA Centered Regulatory Network in Foxtail Millet (Setaria italica L.)
Source: Front Plant Sci. 2022 May 6;13:848474. doi: 10.3389/fpls.2022.848474 (PMC9121102; doi:10.3389/fpls.2022.848474)

T=Seita.9G175400.1_Q=Sit−miR2275b_S=1639

category=2_p=0.999885907436933

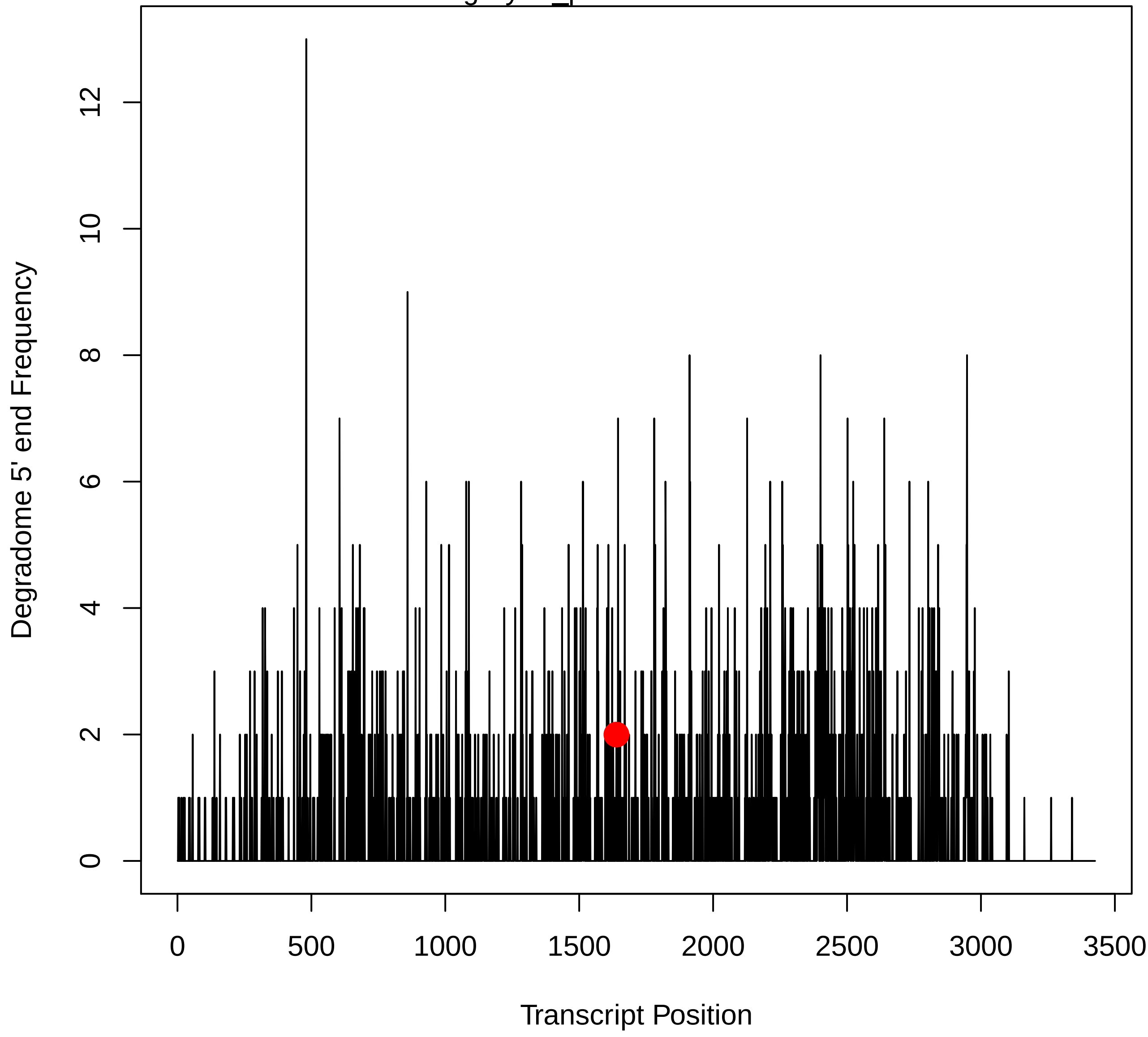

Supplement: Supplementary file 2 [file Data_Sheet_2.zip › Sit-miR2275b_Seita.9G175400.1_1639_TPlot.pdf]
